# Intelligent quality assessment of ultrasound images for fetal nuchal translucency measurement during the first trimester of pregnancy based on deep learning models

**DOI:** 10.1186/s12884-025-07863-y

**Published:** 2025-07-10

**Authors:** Lu Liu, Ting Wang, Wenjing Zhu, Haidong Zhang, Hongyan Tian, Yanping Li, Wenjun Cai, Peng Yang

**Affiliations:** 1 Department of Ultrasound Medicine, Medical School, South China Hospital, Shenzhen University, Shenzhen, P. R. China; 2 Medical Research Department, Qingdao Hospital, University of Health and Rehabilitation Sciences (Qingdao Municipal Hospital), Qingdao, P. R. China; 3 Department of Ultrasound, Medical School, Shenzhen University General Hospital, Shenzhen University, Shenzhen, P. R. China; 4 Guangdong Key Laboratory for Biomedical Measurements and Ultrasound Imaging, School of Biomedical Engineering, National-Regional Key Technology Engineering Laboratory for Medical Ultrasound, Shenzhen University Medical School, Shenzhen University, Shenzhen, P. R. China

**Keywords:** Quality assessment, Nuchal translucency, Deep learning, Vision transformer, Ultrasound standard section

## Abstract

**Objective:**

As increased nuchal translucency (NT) thickness is notably associated with fetal chromosomal abnormalities, structural defects, and genetic syndromes, accurate measurement of NT thickness is crucial for the screening of fetal abnormalities during the first trimester. We aimed to develop a model for quality assessment of ultrasound images for precise measurement of fetal NT thickness.

**Method:**

We collected 2140 ultrasound images of midsagittal sections of the fetal face between 11 and 14 weeks of gestation. Several image segmentation models were trained, and the one exhibiting the highest DSC and HD 95 was chosen to automatically segment the ROI. The radiomics features and deep transfer learning (DTL) features were extracted and selected to construct radiomics and DTL models. Feature screening was conducted using the *t*-test, Mann-Whitney *U*-test, Spearman’s rank correlation analysis, and LASSO. We also developed early fusion and late fusion models to integrate the advantages of radiomics and DTL models. The optimal model was compared with junior radiologists. We used SHapley Additive exPlanations (SHAP) to investigate the model’s interpretability.

**Results:**

The DeepLabV3 ResNet achieved the best segmentation performance (DSC: 98.07 ± 0.02%, HD 95: 0.75 ± 0.15 mm). The feature fusion model demonstrated the optimal performance (AUC: 0.978, 95% CI: 0.965–0.990, accuracy: 93.2%, sensitivity: 93.1%, specificity: 93.4%, PPV: 93.5%, NPV: 93.0%, precision: 93.5%). This model exhibited more reliable performance compared to junior radiologists and significantly improved the capabilities of junior radiologists. The SHAP summary plot showed DTL features were the most important features for feature fusion model.

**Conclusion:**

The proposed models innovatively bridge the gaps in previous studies, achieving intelligent quality assessment of ultrasound images for NT measurement and highly accurate automatic segmentation of ROIs. These models are potential tools to enhance quality control for fetal ultrasound examinations, streamline clinical workflows, and improve the professional skills of less-experienced radiologists.

**Supplementary Information:**

The online version contains supplementary material available at 10.1186/s12884-025-07863-y.

## Introduction

Performing a routine ultrasound examination between 11 and 14 weeks of gestation is valuable for confirming viability and the number of fetuses, accurate pregnancy dating, detecting major fetal abnormalities, and assessing the risk of aneuploidy by measuring fetal nuchal translucency (NT) thickness [1]. As increased NT thickness is notably associated with fetal chromosomal abnormalities, structural defects, and genetic syndromes [2–9], ultrasound measurement of NT thickness has become a crucial component of the combined first-trimester screening model for common aneuploidies [10, 11]. Given that NT thickness is used for risk stratification and may influence the subsequent management of the fetus, precise measurement is essential [12].

To ensure the accuracy of NT measurement, the International Society of Ultrasound in Obstetrics and Gynecology (ISUOG) has described the standard section for NT measurement (enlarged midsagittal view of the fetal face) in detail [1, 12, 13]. Moreover, NT measurements should be performed by personnel who have undergone strict training and committed to participate in continuous quality assurance processes [12]. However, due to the subjectivity, interobserver variability, and lack of experienced operators, obtaining standard section and accurately measuring NT thickness remain a challenge. As performing measurement on non-standard sections can significantly impact the accuracy of NT thickness, it is crucial to conduct a rigorous quality assessment of the ultrasound images and ascertain whether they meet standard criteria.

Nonetheless, quality assessment is both labor-intensive and time-consuming, making it difficult to implement, particularly in regions with limited medical resources. Therefore, an intelligent approach is urgently needed to achieve the automation of quality assessment. Artificial intelligence (AI) technology has made remarkable progress in the field of medical imaging analysis, including obstetric ultrasound [14]. AI models have demonstrated the ability to match or even surpass human performance in tasks such as image segmentation, detection, and classification [15, 16]. Deep learning is considered the foremost AI tool in image analysis [17]. The most commonly used deep learning tool is the convolutional neural network (CNN). Recently, the Vision Transformer (ViT), a type of deep neural network mainly based on the self-attention mechanism, has emerged as a cutting-edge algorithm that could replace or combine traditional techniques [18, 19]. Compared to CNNs, the ViT model comprehends the global relationships among features and trains data with reduced computational resources [18]. However, ViT model is currently seldom applied in the field of fetal ultrasound analysis.

Several studies have been published on developing AI models for analyzing the midsagittal ultrasound section of the fetal face during early pregnancy [20]. However, these studies primarily concentrated on the detection of fetal facial anatomical structures or standard sections [21–24], automatic measurement of fetal biometry [21, 25], and identification of abnormal fetuses [26–28]. Few researches focused on the quality assessment of standard sections for NT measurement and automatic image segmentation [20]. To address the shortage of medical resources, reduce the burden of radiologists on quality assessment, and assist inexperienced radiologists in improving their professional expertise, we innovatively constructed a fusion model that integrated the advantages of radiomics, CNN, and ViT for intelligent quality assessment of ultrasound images for NT measurement. We used the SHapley Additive exPlanations (SHAP) method to investigate the interpretability and visualization of the model. Additionally, we established a deep learning-based image segmentation model to automatically delineate the region of interest (ROI), thereby replacing manual annotation.

## Methods

**Fig. 1:**
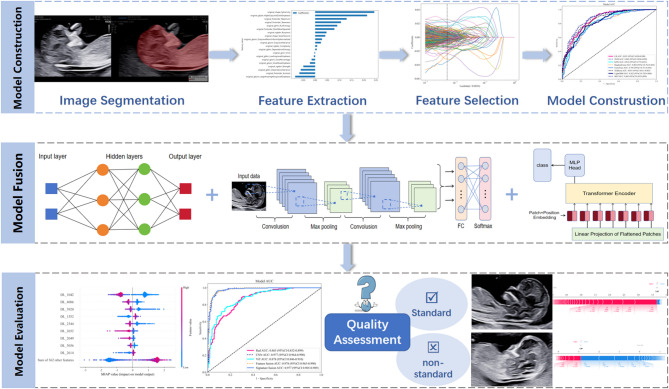
The workflow of the AI model construction

This study received ethical approval from the South China Hospital of Shenzhen University (approval number: HNLS20240326001-A). Given the retrospective nature of the research, the necessity for patient informed consent was not required. Figure 1 depicts the workflow of the AI model construction. Firstly, we trained and developed an image segmentation model to automatically segment the ROIs of the included ultrasound images. Secondly, radiomics features and deep learning features were extracted from the ROIs separately. Thirdly, we employed various methods to select the most significant features for classification. Fourthly, the selected radiomics and deep learning features were input into several machine learning models to construct radiomics models and deep learning models, respectively. Fifthly, we used different indices to evaluate the performance of these models and identify the optimal one. Finally, the SHAP method was utilized to interpret and visualize the model. All models were developed in the same environment (GPU: intel i9 14900KF + NVIDIA RTX 4090D, Memory: 64GB).

### Patients and data acquisition

We retrospectively collected the stored ultrasound images of midsagittal sections of the fetal face in the first trimester of pregnancy at the South China Hospital of Shenzhen University (training cohort) and the Shenzhen University General Hospital (test cohort). The ultrasound examinations were performed between January 2022 and July 2024 using diverse ultrasonographic devices equipped with three-dimensional transabdominal probes, including GE Voluson E8, GE Voluson E10, and Samsung HERA XW10. In accordance with the requirements for NT measurement specified in international authoritative obstetric ultrasound practice guidelines [1, 12], two senior radiologists (W.C. and H.Z.) with over 15 years of experience in obstetric ultrasound classified the included images into standard sections and non-standard sections. Their consistent classifications served as the standard to assess the performance of the models and junior radiologists. In cases where their classifications of an image differed, they would seek a resolution by consulting with a third senior radiologist (H.T. with over 20 years of experience in obstetric ultrasound).

The inclusion criteria were as follows: (1) Pregnant women underwent ultrasound examinations between 11 and 14 weeks of gestation at the two hospitals specified above; (2) Fetal crown-rump length between 45 and 84 mm; (3) Singleton pregnancy; (4) It is routine practice to acquire multiple midsagittal sections of the face for each fetus to obtain the maximum NT thickness. However, only one image per fetus, which exhibits the highest image quality, was selected for further analysis. Two senior radiologists (L.L. and H.T.) performed the image quality control and selection. The exclusion criteria were as follows: (1) Multiple pregnancy; (2) Fetuses diagnosed with severe cranial or facial malformations (such as exencephaly, anencephaly, encephaloceles, absence of the nasal bone, and so on); (3) Ultrasound images with low quality or with measurement lines.

Images meeting all the following criteria were considered to be the standard sections for NT measurement [1, 12]:

1. The image should be magnified to include only the fetal head and upper thorax;
2. NT and nasal bone should be visible;
3. The fetus in a neutral position (i.e. neither flexed nor hyperextended);
4. To ensure that the fetus is not flexed, amniotic fluid should be visible between the fetal chin and chest;
5. Midsagittal view of fetal face and proper alignment of the neck with the trunk;
6. The midsagittal view of the fetal face is defined by the presence of the echogenic nasal tip and rectangular shape of the palate anteriorly, the translucent diencephalon in the center, and the nuchal membrane posteriorly;
7. The orthogonal osseous extension at the frontal end of the maxilla should not be visible.

### Construction of image segmentation model

In training cohort, 250 images were randomly selected for manual delineation and segmentation model training. Two radiologists (L.L. and W.C.) utilized Labelme software (Version 5.3.1, USA) to manually delineate the ROIs, encompassing the fetal head and upper thorax, within the chosen images. The manual annotation served as the training set for constructing the segmentation model and as the reference standard for evaluating the model’s performance. A precisely annotated training set is an important prerequisite for improving the performance of the segmentation model. To avoid the subjective biases arising from personal variations, the interclass correlation coefficient (ICC) was applied to measure the agreement between observers or within the same observer. An ICC value of 0.75 or above was considered to indicate satisfactory agreement.

Several deep learning-based models for image segmentation were trained, such as FCN ResNet, DeepLabV3 ResNet, DeepLabV3 MobileNetV3-Large, LR-ASPP MobileNetV3-Large, and U-Net. The Dice similarity coefficient (DSC) was employed to quantify the overlap between the segmentation outcomes of the radiologists and the segmentation model, thereby assessing the accuracy of segmentation. Additionally, the 95th percentile Hausdorff distance (HD 95), as a contour-based metric, was also utilized to evaluate segmentation consistency. The model exhibiting the highest DSC and HD 95 was chosen to automatically segment the remaining images, and any inaccuracies in segmentation were manually adjusted for precision.

### Feature extraction

The radiomics features were extracted using the Pyradiomics analysis program, an online tool dedicated to radiomics analysis (http://pyradiomics.readthedocs.io). These features can be divided into three primary groups: geometry, intensity, and texture features. Geometry features provide insights into the shape properties of the ROI. Intensity features offer a representation of the first-order statistical distribution of voxel intensities within the ROI. Texture features elucidate the patterns or the second- and higher-order spatial distributions of the intensities.

The deep learning features were extracted from 33 types of networks derived from 10 series of deep learning models, including 9 series of CNN (ResNet, VGG, DenseNet, MobileNet, Inception, SqueezeNet, ShuffleNetV2, MNASNet, and AlexNet) and ViT model. Due to the limited training data, deep learning models frequently suffer from overfitting. To address this issue, we applied transfer learning techniques to pre-train the models with images from the ImageNet database. Then, the parameters of these pre-trained deep transfer learning (DTL) models were utilized to extract deep learning features. Usually, the output features of the penultimate fully connected layer were extracted as deep learning features.

### Feature selection and dimensionality reduction

All features were normalized using the Z-score method, resulting in a standard normal distribution. The *t*-test or Mann-Whitney *U*-test was used for feature screening. To select the features that demonstrated significant differences between standard and non-standard sections, only those features with a *P* value of less than 0.05 were retained. To address the issue of high repeatability among features, we used Spearman’s rank correlation coefficient to ascertain the correlation between them and assess their multicollinearity. We retained only one feature from any pair that exhibited a correlation coefficient exceeding 0.9, thereby eliminating those characterized by high repeatability.

We utilized the least absolute shrinkage and selection operator (LASSO) regression model, as implemented in the scikit-learn package for Python (version 3.70), to perform feature selection and dimensionality reduction. According to the regulation weight λ, LASSO reduced all regression coefficients towards zero and precisely set the coefficients of the irrelevant features to zero. The optimal λ was determined using 10-fold cross-validation with a minimal criteria approach, where the final value of λ led to the minimum cross-validation error. The most robust non-redundant retained features with non-zero coefficients were selected to establish the Rad score. Ultimately, a Rad score for each image was derived through a linear combination of the retained features, each weighted by their respective model coefficients.

### Model construction and performance evaluation

The selected radiomics or DTL features were input into several machine learning models, such as Logistic Regression (LR), Support Vector Machine (SVM), k-nearest neighbor (KNN), Random Forest, Extra Trees, XGBoost, LightGBM, and Multi-Layer Perception (MLP), to construct the classification models using the scikit-learn package in Python (version 3.70). Following this, 5-fold cross-verification was performed to ascertain the optimal model hyperparameters for model fitting, and the most robust and non-redundant signatures were derived for each model.

To assess the effectiveness of the models’ classification abilities, receiver operating characteristic (ROC) curves were generated to evaluate the classification performance visually. Furthermore, several indices were calculated, including area under the ROC curve (AUC), specificity, sensitivity, accuracy, positive predictive value (PPV), negative predictive value (NPV), and precision. The DeLong test was performed to compare the AUCs of different models. To evaluate the consistency between the predicted probabilities of the models and the actual classification probabilities, calibration curves were drawn and the calibration efficiency was assessed using Hosmer-Lemeshow analysis. Additionally, decision curve analysis (DCA) was conducted to assess the clinical utility of the predictive models.

### Construction of fusion models

To improve the accuracy of classification, we developed fusion models that integrated the advantages of radiomics, CNN, and ViT to derive the optimal model. Based on the stage of the data processing workflow where fusion occurs, the fusion strategies can be categorized into early fusion (feature fusion) and late fusion (signature fusion). Early fusion typically refers to the process of merging features from different sources into a unified dataset at the input stage of the model. The advantage of early fusion lies in its ability to capture the correlations between different data sources. Feature fusion was achieved by concatenating the extracted radiomics features, CNN features, and ViT features. The fused features then underwent the processes of feature screening to select the most important features for classification and model construction as described above, resulting in the establishment of the feature fusion model.

Within the framework of late fusion, each modality’s data generates prediction results through separate models, and these independent results are then combined to form the final output. Late fusion can be implemented in various ways, such as through voting mechanisms, weighted averaging, stacking ensemble, or by utilizing another machine learning model to synthesize the results produced by different models. The advantage of this approach is that each model can focus on extracting the most relevant information from its modality without being influenced by others. In this study, signature fusion (i.e. result fusion) was realized by independently developing separate radiomics model, CNN model, and ViT model. The output results of these models were represented as signatures. Subsequently, the signatures of each individual model were integrated using a stacking ensemble strategy to construct the signature fusion model [29].

### Radiologists-AI model comparison

According to the guidelines, two senior radiologists (W.C. and H.Z.) independently classified all the included ultrasound images into standard and non-standard sections. Two junior radiologists (T.W. with less than 5 years of experience in obstetric ultrasound and Y. L. with less than 3 years of experience in obstetric ultrasound) were tasked to classify the images in test cohort. Using the classification of senior radiologists as the standard, we compared the classification performance of the junior radiologists and the AI model. The junior radiologists were directed to reclassify each image with the assistance of the AI model and were evaluated whether the classification accuracy was improved.

### Model interpretability and visualization

To illustrate the significance of the features and their influence on the overall classification model, we employed the SHAP method, derived from game theory, to visualize the decision-making process of machine learning models. This approach enables the identification and prioritization of features that determine compound classification. The model demonstrating the optimal classification performance was investigated using SHAP method.

### Statistical analysis

The continuous variables, such as the age of pregnancy women and gestational age of fetuses, were described using mean ± standard deviation (SD) and analyzed with *t*-test or Mann–Whitney *U*-test using IBM SPSS (version 21.0). Statistical significance was determined by a two-sided *P*-value < 0.05. The 95% confidence interval (CI) of the AUC was also calculated. Furthermore, Python (version 3.70) was utilized to conduct ICCs, Spearman rank correlation tests, Z-score normalization, and LASSO regression analysis.

## Results

### Clinical characteristics

**Fig. 2:**
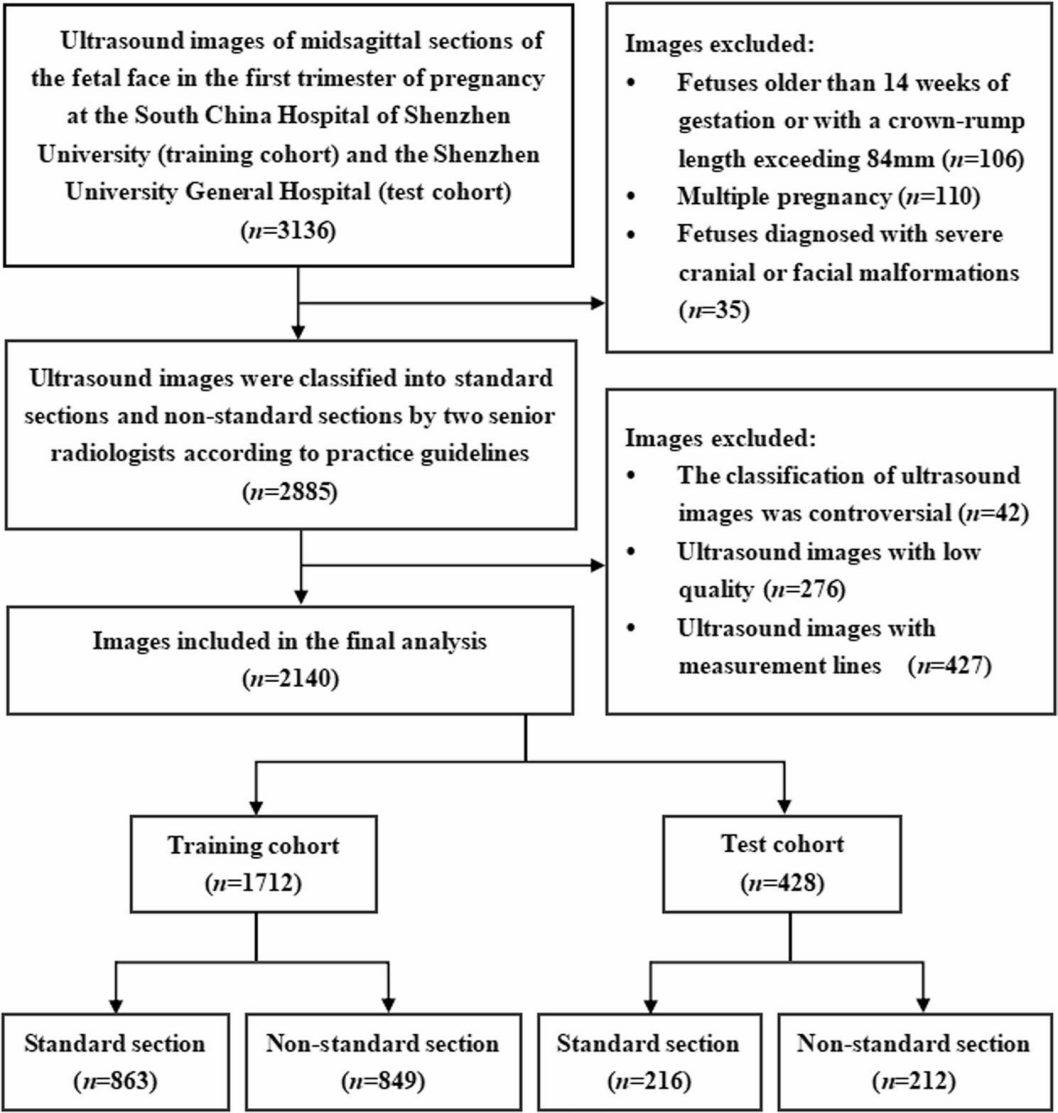
Flowchart of the study subjects screening based on inclusion and exclusion criteria

A total of 2140 pregnant women, each providing an ultrasound image, were enrolled in the study. Of these, 1712 cases were from the training cohort and 428 cases were from the test cohort, adhering to a radio of 8:2. The mean age of the training cohort and test cohort was 30.53 ± 6.81 years and 32.25 ± 7.33 years, respectively (*P* = 0.086). The median gestational age in the training cohort and test cohort was 12 + 6 weeks and 13 + 0 weeks, respectively (*P* = 0.347). No significant differences were observed in the mean age and median gestational age between the two cohorts (*P* > 0.05). Based on the classification by senior radiologists, in the training cohort, 863 images (50.41%) were classified as standard sections, while 849 images (49.59%) as non-standard sections. In the test cohort, 216 images (50.47%) were identified as standard, whereas 212 images (49.53%) were considered non-standard (Fig. 2). There was no statistical difference in the distribution of standard and non-standard sections between the training and test cohorts (*P* > 0.05). Table 1 provides the baseline characteristics of the two cohorts.

**Table 1:** Baseline clinical characteristics of participants between two cohorts

Clinical features	Training cohort	Test cohort	*P*
No. of pregnant women	1712	428	/
No. of ultrasound images for NT measurement	1712	428	/
Age (Mean ± SD, years)	30.53 ± 6.81	32.25 ± 7.33	0.086
Median gestational age	12 + 6 weeks	13 + 0 weeks	0.347
No. of standard sections	863 (50.41%)	216 (50.47%)	0.756
No. of non-standard sections	849 (49.59%)	212 (49.53%)	0.728

### Performance of image segmentation models

**Fig. 3:**
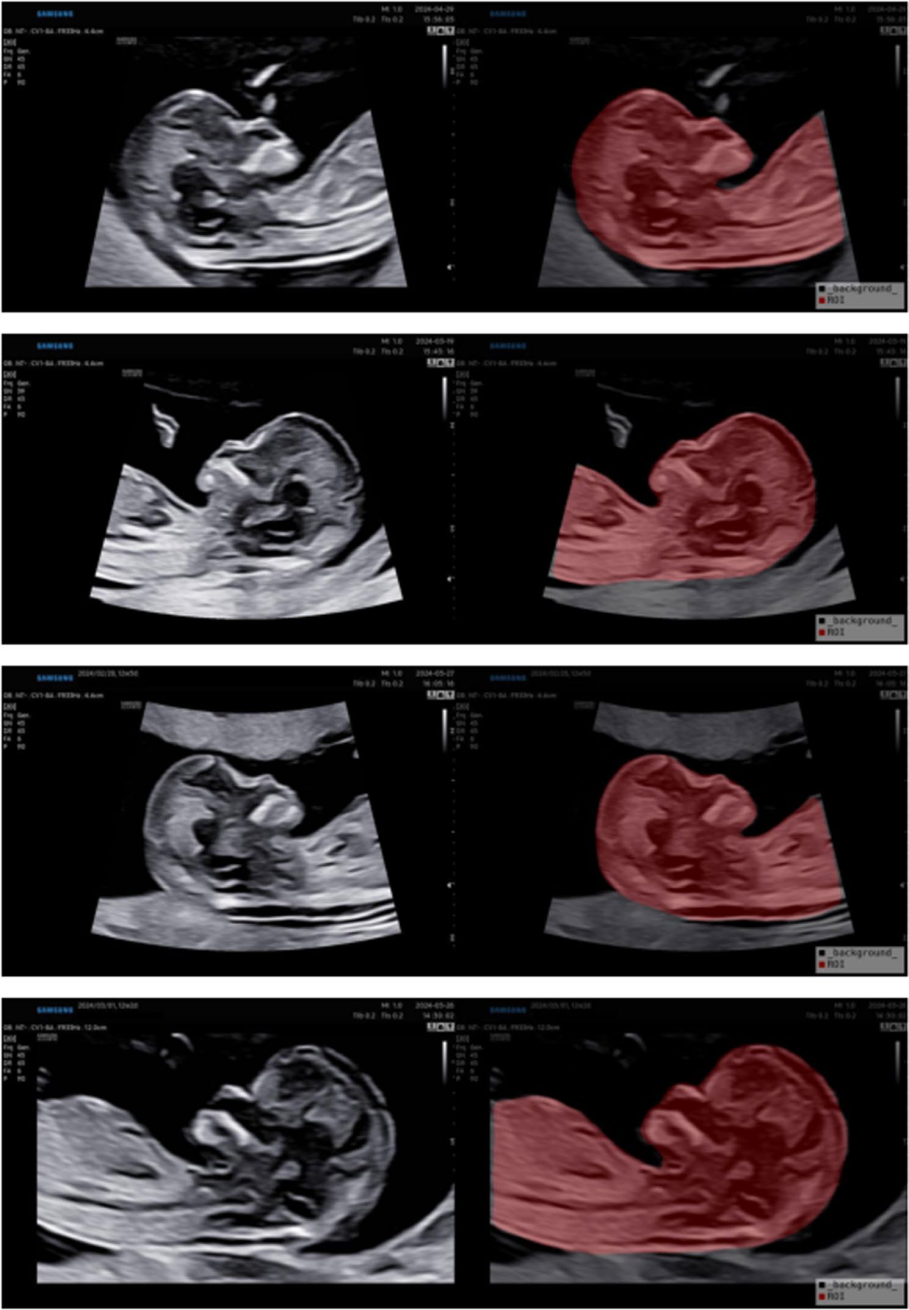
The segmentation results of the automatic segmentation model DeepLabV3 ResNet

The DeepLabV3 ResNet model achieved the best image segmentation performance (DSC: 98.07 ± 0.02%, HD 95: 0.75 ± 0.15 mm). Therefore, this model was utilized to automate the segmentation of the remaining images in both the training and test cohorts. The mean duration for automatic segmentation of a single image was 3.5 s in this model. In contrast, manual annotation required an average of 14.1 s per image. This segmentation model enabled a 75.2% reduction in the time required for image segmentation. Table 2 presents the DSC and HD 95 of deep learning-based segmentation models. Figure 3 illustrates the segmentation results generated by the DeepLabV3 ResNet model.

**Table 2:** DSC and HD 95 of different deep learning-based segmentation models compared with the annotations from two radiologists

Model	FCN ResNet	DeepLabV3 ResNet	DeepLabV3 MobileNetV3-Large	LR-ASPP MobileNetV3-Large	U-Net
DSC (Mean ± SD, %)	97.95 ± 0.08	98.07 ± 0.02	96.26 ± 0.02	95.31 ± 0.02	93.91 ± 0.02
HD 95 (Mean ± SD, mm)	0.82 ± 0.21	0.75 ± 0.15	0.93 ± 0.11	1.03 ± 0.12	1.23 ± 0.14

### Feature extraction and selection

**Fig. 4:**
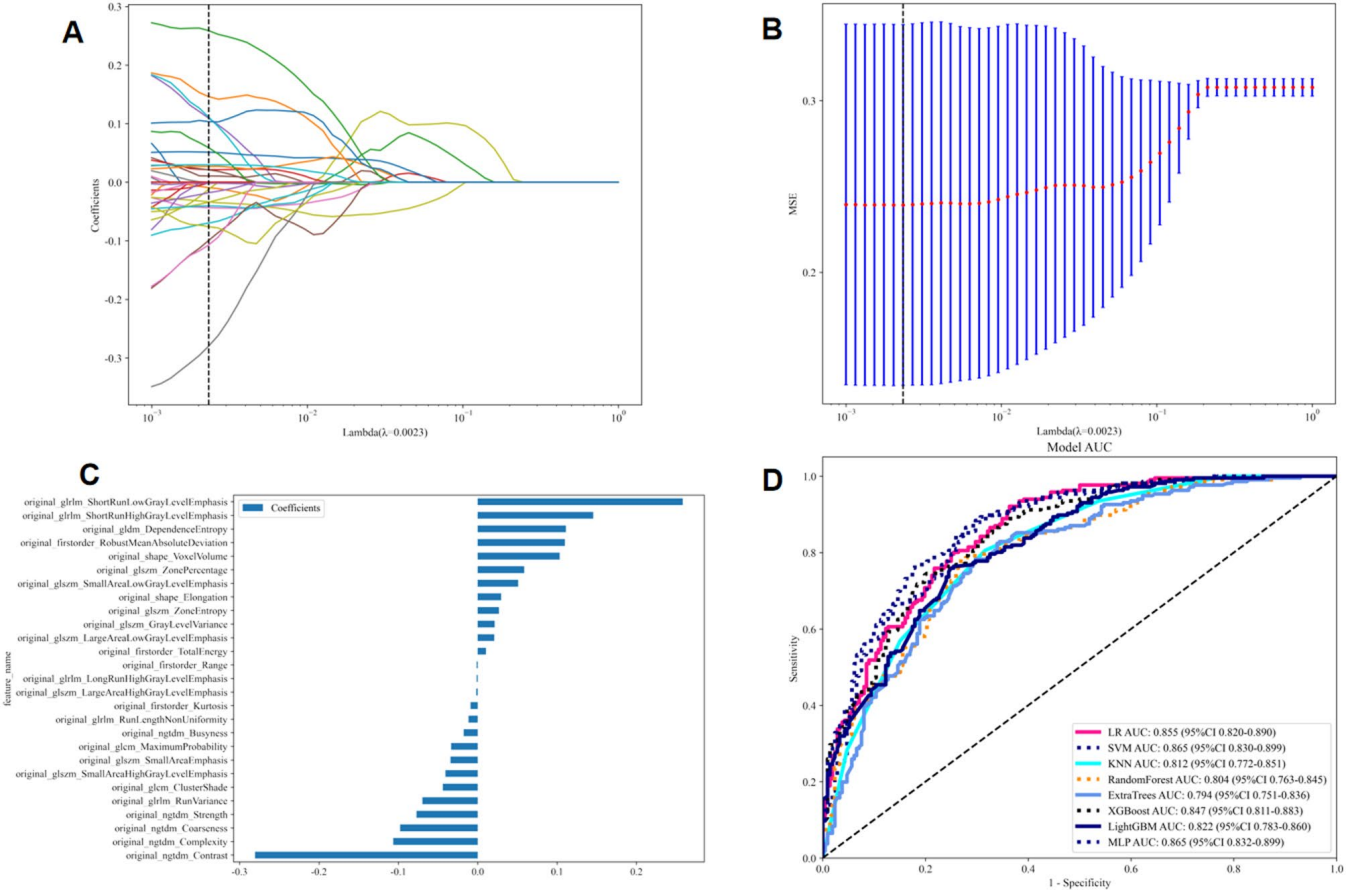
Radiomics feature selection using the LASSO logistic regression in test cohort. **A** Coefficients of 10-fold cross-validation based on LASSO. **B** MSE of 10-fold cross-validation based on LASSO. **C** Histogram depicting the values of coefficients in the final selected non-zero features. **D** The ROC curves of radiomics model. MSE: mean square error

In the study, 107 handcrafted radiomics features were extracted, which comprised 14 shape features, 18 first-order features, and 75 texture features. Additionally, we pre-trained 33 types of DTL models, including CNN and ViT models, to extract between 338 and 16,383 DTL features, depending on the models. The Spearman rank correlation test and the LASSO logistic regression were used for feature selection and dimensionality reduction. Details of the extraction and selection of radiomics features and DTL features are presented in Figs. 4A, B, and C and 5A, B, C, and D.

**Fig. 5:**
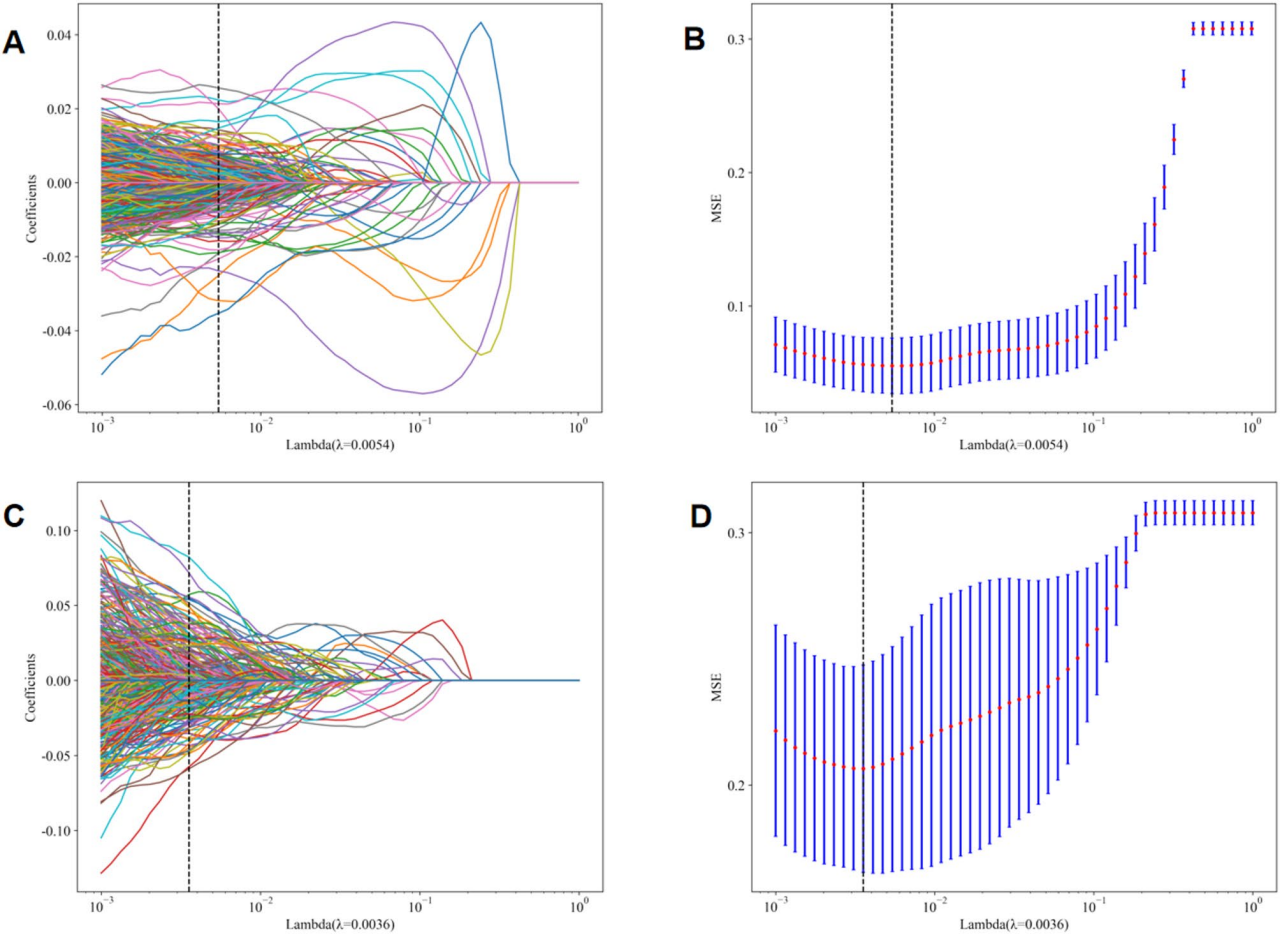
TL feature selection using the LASSO logistic regression in test cohort. **A** DTL (VGG19_bn model) feature selection: Coefficients of 10-fold cross-validation based on LASSO. **B** DTL (VGG19_bn model) feature selection: MSE of 10-fold cross-validation based on LASSO. **C** DTL (ViT model) feature selection: Coefficients of 10-fold cross-validation based on LASSO. **D** DTL (ViT model) feature selection: MSE of 10-fold cross-validation based on LASSO. MSE: mean square error

### Performance of the radiomics model

Eight machine learning models were developed and evaluated to identify the optimal model for classification. In the test cohort, MLP algorithm demonstrated superior performance (AUC: 0.865, 95% CI: 0.832–0.899, accuracy: 79.2%, sensitivity: 88.0%, specificity: 70.3%, PPV: 75.1%, NPV: 85.1%, precision: 75.1%) (Fig. 4D). Table 3 presents the evaluation indices of different radiomics models in training and test cohorts. The hyperparameter details for each model are presented in Supplementary Material 1.

**Table 3:** Classification performance of different machine learning algorithms for radiomics models

Algorithm	Cohort	AUC (95% CI)	Accuracy (%)	Sensitivity (%)	Specificity (%)	PPV (%)	NPV (%)	Precision (%)
LR	Training	0.857 (0.839–0.874)	77.7	80.8	74.6	76.3	79.2	76.3
LR	Test	0.855 (0.821–0.890)	77.8	91.7	63.7	72.0	88.2	72.0
SVM	Training	0.900 (0.886–0.915)	82.1	86.0	78.2	80.0	84.6	80.0
SVM	Test	0.865 (0.830–0.899)	79.2	76.4	82.1	81.3	77.3	81.3
KNN	Training	0.899 (0.886–0.913)	79.2	66.5	92.1	89.5	73.0	89.5
KNN	Test	0.812 (0.772–0.851)	70.8	56.9	84.9	79.4	65.9	79.4
Random Forest	Training	0.818 (0.798–0.837)	74.5	76.9	72.1	73.7	75.5	73.7
Random Forest	Test	0.804 (0.763–0.845)	75.5	77.8	73.1	74.7	76.4	74.7
Extra Trees	Training	0.790 (0.769–0.811)	72.1	73.8	70.4	71.7	72.6	71.7
Extra Trees	Test	0.794 (0.752–0.836)	74.1	77.8	70.3	72.7	75.6	72.7
XGBoost	Training	0.878 (0.862–0.893)	79.0	72.5	85.5	83.6	75.4	83.6
XGBoost	Test	0.847 (0.812–0.883)	76.9	73.6	80.2	79.1	74.9	79.1
LightGBM	Training	0.847 (0.829–0.864)	76.0	77.2	74.8	75.7	76.3	75.7
LightGBM	Test	0.822 (0.783–0.860)	75.2	75.0	75.5	75.7	74.8	75.7
MLP	Training	0.894 (0.879–0.909)	81.4	87.3	75.5	78.4	85.4	78.4
MLP	Test	0.865 (0.832–0.899)	79.2	88.0	70.3	75.1	85.1	75.1

### Performance of DTL models

**Fig. 6:**
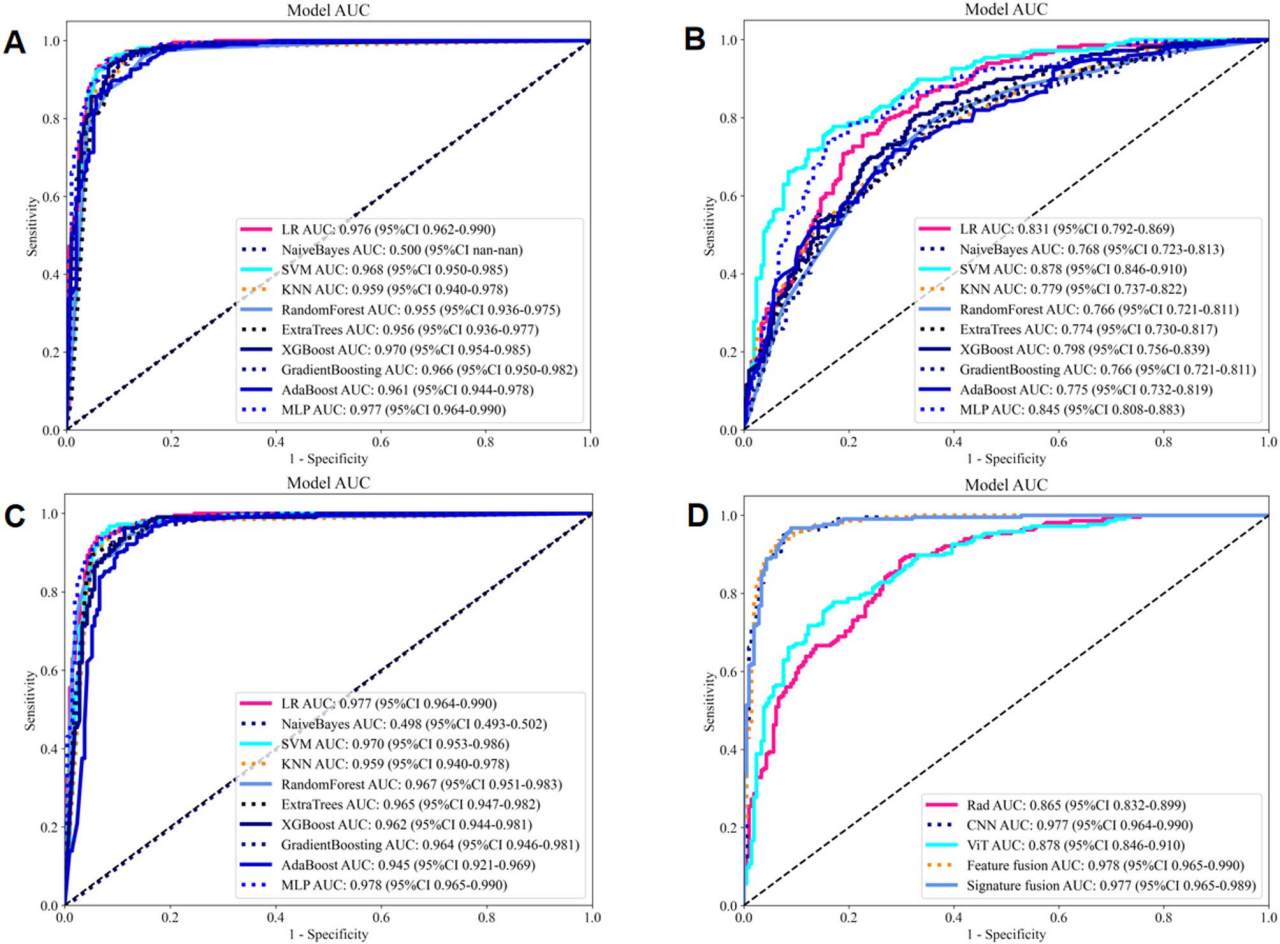
The ROC curves of different models in test cohort. **A** The ROC curves of CNN model (VGG19_bn). **B** The ROC curves of ViT model. **C** The ROC curves of feature fusion model. **D** The ROC curves of radiomics model, CNN model (VGG19_bn), ViT model, feature fusion model, and signature fusion model

Several machine learning models were developed using the selected DTL features. The pre-trained VGG19_bn model with the MLP algorithm achieved the highest performance in test cohort (AUC: 0.977, 95% CI: 0.964–0.990, accuracy: 93.5%, sensitivity: 94.4%, specificity: 92.5%, PPV: 92.7%, NPV: 94.2%, precision: 92.7%) (Fig. 6A). In addition, the AUC, accuracy, sensitivity, specificity, PPV, NPV, and precision of the pre-trained ViT model with SVM algorithm was 0.878 (95% CI: 0.846–0.910), 80.1%, 77.3%, 83.0%, 82.3%, 78.2%, and 82.3%, respectively (Fig. 6B). Table 4 presents the evaluation indices of different DTL models in training and test cohorts.

**Table 4:** The best classification performance of different DTL models

Model Series	Model Name	Cohort	AUC (95% CI)	Accuracy (%)	Sensitivity (%)	Specificity (%)	PPV (%)	NPV (%)	Precision (%)
ResNet	ResNet18	Training	0.999 (0.999-1.000)	99.1	99.0	99.3	99.3	98.9	99.3
	ResNet18	Test	0.970 (0.955–0.985)	93.0	94.4	91.5	91.9	94.2	91.9
	ResNet50	Training	1.000 (0.999-1.000)	99.4	99.1	99.8	99.8	99.1	99.8
	ResNet50	Test	0.975 (0.962–0.987)	92.1	92.1	92.0	92.1	92.0	92.1
	ResNet101	Training	1.000 (1.000–1.000)	99.9	99.9	100.0	100.0	99.9	100.0
	ResNet101	Test	0.972 (0.959–0.986)	92.1	94.0	90.1	90.6	93.6	90.6
	ResNet152	Training	1.000 (0.999-1.000)	99.6	99.8	99.5	99.5	99.8	99.5
	ResNet152	Test	0.968 (0.953–0.983)	91.8	93.5	90.1	90.6	93.2	90.6
	ResNeXt50_32 × 4d	Training	1.000 (0.999-1.000)	99.5	99.8	99.3	99.3	99.8	99.3
	ResNeXt50_32 × 4d	Test	0.966 (0.950–0.982)	91.6	92.6	90.6	90.9	92.3	90.9
	ResNeXt101_32 × 8d	Training	1.000 (1.000–1.000)	99.9	99.9	100.0	100.0	99.9	100.0
	ResNeXt101_32 × 8d	Test	0.971 (0.958–0.984)	91.6	92.1	91.0	91.3	91.9	91.3
	Wide_ResNet50_2	Training	1.000 (0.999-1.000)	99.8	99.7	100.0	100.0	99.6	100.0
	Wide_ResNet50_2	Test	0.965 (0.949–0.982)	92.3	92.1	92.5	92.6	92.0	92.6
	Wide_ResNet101_2	Training	1.000 (0.999-1.000)	99.8	99.7	99.9	99.9	99.6	99.9
	Wide_ResNet101_2	Test	0.957 (0.939–0.975)	90.0	92.1	87.7	88.4	91.6	88.4
VGG	VGG11	Training	0.999 (0.998-1.000)	98.7	98.0	99.4	99.4	98.0	99.4
	VGG11	Test	0.971 (0.957–0.984)	90.9	90.7	91.0	91.2	90.6	91.2
	VGG11_bn	Training	0.996 (0.994–0.998)	97.0	96.9	97.2	97.2	96.8	97.2
	VGG11_bn	Test	0.968 (0.953–0.982)	91.1	88.4	93.9	93.6	88.8	93.6
	VGG13	Training	1.000 (0.999-1.000)	99.8	99.7	100.0	100.0	99.6	100.0
	VGG13	Test	0.962 (0.945–0.978)	90.7	94.0	87.3	88.3	93.4	88.3
	VGG13_bn	Training	1.000 (1.000–1.000)	99.9	99.9	99.9	99.9	99.9	99.9
	VGG13_bn	Test	0.967 (0.952–0.983)	91.8	92.1	91.5	91.7	91.9	91.7
	VGG16	Training	1.000 (0.999-1.000)	99.7	99.5	99.9	99.9	99.5	99.9
	VGG16	Test	0.966 (0.949–0.982)	91.6	91.7	91.5	91.7	91.5	91.7
	VGG16_bn	Training	1.000 (1.000–1.000)	99.9	99.9	100.0	100.0	99.9	100.0
	VGG16_bn	Test	0.965 (0.948–0.982)	92.5	90.3	94.8	94.7	90.5	94.7
	VGG19	Training	1.000 (1.000–1.000)	99.6	99.7	99.5	99.5	99.6	99.5
	VGG19	Test	0.970 (0.956–0.984)	91.6	89.4	93.9	93.7	89.6	93.7
	VGG19_bn	Training	1.000 (1.000–1.000)	99.9	99.9	99.9	99.9	99.9	99.9
	VGG19_bn	Test	0.977 (0.964–0.990)	93.5	94.4	92.5	92.7	94.2	92.7
DenseNet	DenseNet121	Training	1.000 (1.000–1.000)	99.8	99.7	100.0	100.0	99.6	100.0
	DenseNet121	Test	0.967 (0.953–0.982)	90.9	89.8	92.0	91.9	89.9	91.9
	DenseNet161	Training	1.000 (0.999-1.000)	99.5	99.2	99.9	99.9	99.2	99.9
	DenseNet161	Test	0.963 (0.945–0.981)	91.1	89.8	92.5	92.4	89.9	92.4
	DenseNet169	Training	1.000 (1.000–1.000)	99.6	99.9	99.3	99.3	99.9	99.3
	DenseNet169	Test	0.973 (0.961–0.985)	91.8	96.8	86.8	88.2	96.3	88.2
	DenseNet201	Training	1.000 (1.000–1.000)	99.8	99.5	100.0	100.0	99.5	100.0
	DenseNet201	Test	0.971 (0.955–0.986)	92.1	90.7	93.4	93.3	90.8	93.3
MobileNet	MobileNet_v2	Training	0.999 (0.998-1.000)	98.8	98.4	99.2	99.2	98.4	99.2
	MobileNet_v2	Test	0.972 (0.959–0.986)	91.8	92.1	91.5	91.7	91.9	91.7
	MobileNet_v3_large	Training	0.985 (0.979–0.990)	94.6	93.3	96.0	95.9	93.4	95.9
	MobileNet_v3_large	Test	0.929 (0.904–0.954)	87.1	90.7	83.5	84.8	89.8	84.8
	MobileNet_v3_small	Training	0.982 (0.977–0.987)	93.5	94.7	92.3	92.6	94.5	92.6
	MobileNet_v3_small	Test	0.911 (0.883–0.939)	85.5	84.7	86.3	86.3	84.7	86.3
Inception	GoogleNet	Training	0.997 (0.994–0.999)	98.1	98.7	97.5	97.6	98.7	97.6
	GoogleNet	Test	0.933 (0.910–0.956)	87.1	83.3	91.0	90.5	84.3	90.5
	Inception_v3	Training	0.998 (0.996–0.999)	98.5	98.7	98.2	98.3	98.7	98.3
	Inception_v3	Test	0.965 (0.947–0.983)	92.3	89.8	94.8	94.6	90.1	94.6
SqueezeNet	SqueezeNet1_0	Training	0.992 (0.988–0.996)	96.4	96.2	96.6	96.6	96.1	96.6
	SqueezeNet1_0	Test	0.953 (0.935–0.971)	88.6	86.6	90.6	90.3	86.9	90.3
	SqueezeNet1_1	Training	0.985 (0.979–0.990)	95.0	93.5	96.5	96.4	93.6	96.4
	SqueezeNet1_1	Test	0.932 (0.909–0.955)	85.7	82.9	88.7	88.2	83.6	88.2
ShuffleNetV2	ShuffleNet_v2_x0_5	Training	1.000 (1.000–1.000)	99.9	99.9	100.0	100.0	99.9	100.0
	ShuffleNet_v2_x0_5	Test	0.906 (0.878–0.933)	84.3	81.9	86.8	86.3	82.5	86.3
	ShuffleNet_v2_x1_0	Training	1.000 (1.000–1.000)	99.9	99.9	100.0	100.0	99.9	100.0
	ShuffleNet_v2_x1_0	Test	0.923 (0.899–0.947)	85.3	84.7	85.8	85.9	84.7	85.9
MNASNet	MNASNet0_5	Training	0.996 (0.994–0.998)	97.0	97.5	96.6	96.7	97.4	96.7
	MNASNet0_5	Test	0.880 (0.847–0.913)	83.9	88.0	79.7	81.5	86.7	81.5
	MNASNet1_0	Training	0.996 (0.993–0.999)	98.1	97.6	98.7	98.7	97.6	98.7
	MNASNet1_0	Test	0.892 (0.862–0.922)	82.0	90.3	73.6	77.7	88.1	77.7
AlexNet	AlexNet	Training	1.000 (0.999-1.000)	99.1	98.7	99.5	99.5	98.7	99.5
	AlexNet	Test	0.955 (0.936–0.973)	89.7	89.8	89.6	89.8	89.6	89.8
ViT	ViT	Training	0.982 (0.977–0.987)	93.3	92.2	94.3	94.3	92.3	94.3
	ViT	Test	0.878 (0.846–0.910)	80.1	77.3	83.0	82.3	78.2	82.3

### Performance of fusion models

The radiomics features, pre-trained CNN features (VGG19_bn), and pre-trained ViT features were concatenated to construct feature fusion models and the MLP algorithm showed the best performance in test cohort (AUC: 0.978, 95% CI: 0.965–0.990, accuracy: 93.2%, sensitivity: 93.1%, specificity: 93.4%, PPV: 93.5%, NPV: 93.0%, precision: 93.5%) (Fig. 6C). The signatures of radiomics model, DTL model (VGG19_bn), and ViT model were integrated using stacking ensemble strategy to construct signature fusion models and the LR algorithm demonstrated the highest level of classification performance in test cohort (AUC: 0.977, 95% CI: 0.965–0.989, accuracy: 93.7%, sensitivity: 96.3%, specificity: 91.0%, PPV: 91.6%, NPV: 96.0%, precision: 91.6%). Table 5 presents the evaluation indices of these models in training and test cohorts. The ROC curves of different models in the test cohort are shown in Fig. 6D. 

**Table 5:** Classification performance and computational time of radiomics, CNN, ViT, feature fusion, and signature fusion models

Model	Cohort	AUC (95% CI)	Accuracy (%)	Sensitivity (%)	Specificity (%)	PPV (%)	NPV (%)	Precision (%)	Time (hour)
Radiomics	Training	0.894 (0.879–0.909)	81.4	87.3	75.5	78.4	85.4	78.4	78.5
Radiomics	Test	0.865 (0.832–0.899)	79.2	88.0	70.3	75.1	85.1	75.1	
CNN	Training	1.000 (1.000–1.000)	99.9	99.9	99.9	99.9	99.9	99.9	2.0
CNN	Test	0.977 (0.964–0.990)	93.5	94.4	92.5	92.7	94.2	92.7	
ViT	Training	0.982 (0.977–0.987)	93.3	92.2	94.3	94.3	92.3	94.3	2.0
ViT	Test	0.878 (0.846–0.910)	80.1	77.3	83.0	82.3	78.2	82.3	
Feature fusion	Training	1.000 (1.000–1.000)	99.9	99.9	99.9	99.9	99.9	99.9	2.5
Feature fusion	Test	0.978 (0.965–0.990)	93.2	93.1	93.4	93.5	93.0	93.5	
Signature fusion	Training	1.000 (1.000–1.000)	99.9	99.9	100.0	100.0	99.9	100.0	2.5
Signature fusion	Test	0.977 (0.965–0.989)	93.7	96.3	91.0	91.6	96.0	91.6	

### Performance comparison between radiomics, DTL, and fusion models

Among the radiomics, CNN, ViT, and fusion models, the feature fusion model demonstrated the optimal classification performance (Table 5; Fig. 6D). The pre-trained CNN features (VGG19_bn) were used in the feature fusion. Extracting radiomic features was a time-consuming process, resulting in the radiomics model having the longest computation time (78.5 h). However, the fusion model, which utilized the pre-extracted radiomic features from the radiomics model construction, experienced significantly reduced computation time (2.5 h). The DTL model had the shortest computational time (2.0 h) (Table 5).

**Fig. 7:**
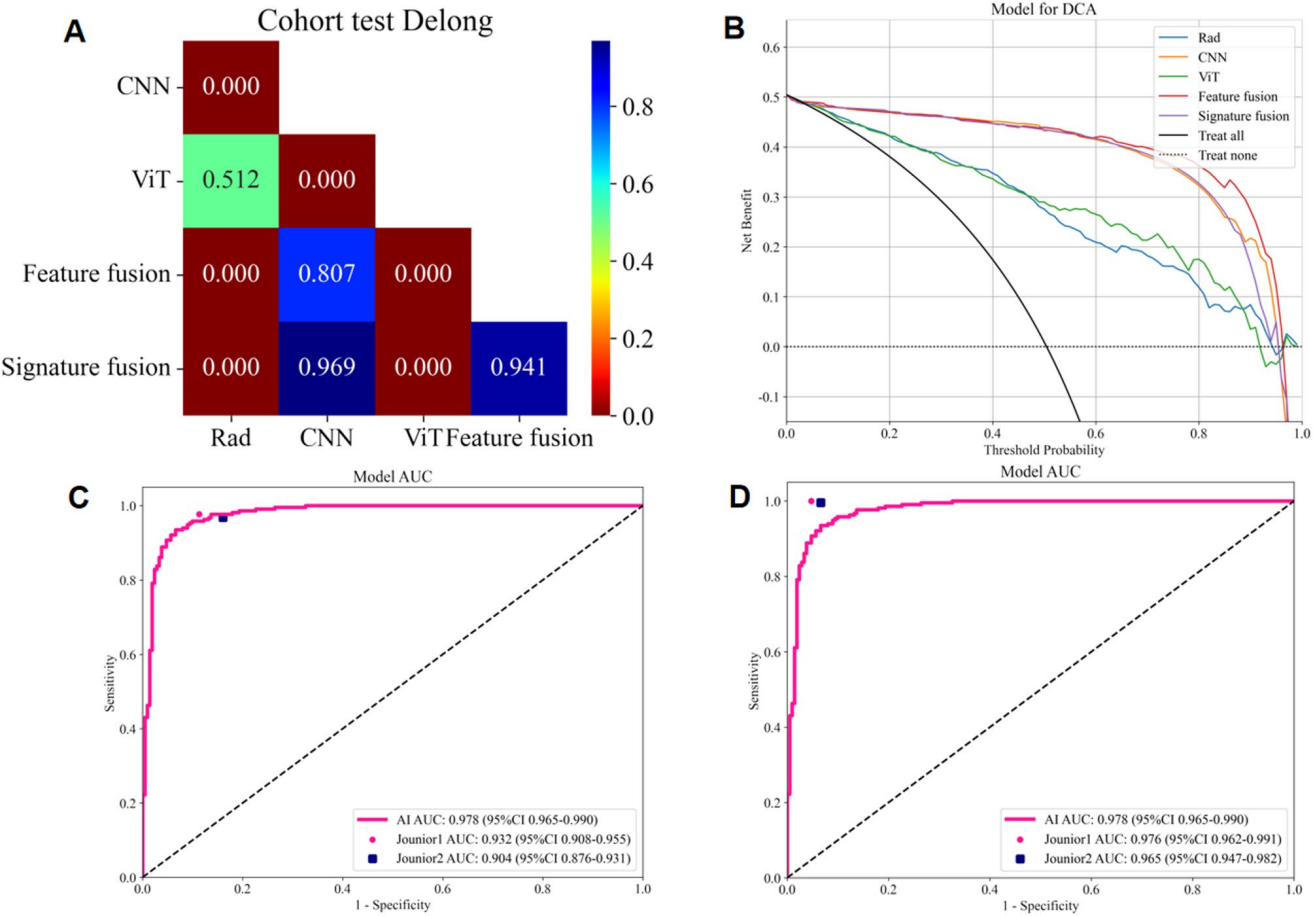
**A** The *P*-value of DeLong test between different models in test cohort; **B** The DCA curves for different models in test cohort; **C** The ROC curves for junior radiologists without the assistance of the AI model; **D** The ROC curves for junior radiologists with the assistance of the AI model

The DeLong test indicated that in the test cohort, the comparison of AUC between the radiomics and CNN models, between the radiomics and fusion models, between the CNN and ViT models, as well as between the ViT and fusion models, was statistically significant (*P* < 0.05). However, there was no statistically significant difference between the CNN, feature fusion, and signature fusion models, as well as between the radiomics and ViT models (*P* > 0.05). The findings suggested that the CNN, feature fusion, and signature fusion models outperformed the radiomics and ViT models in terms of classification performance. Figure 7A presents the *P*-value of the DeLong test between different models. In the test cohort, the Hosmer-Lemeshow test revealed good consistency between the predicted probabilities of all models and the actual classification (*P* > 0.05), indicating that these models were well-calibrated. The analysis of the DCA curve illustrated that all models substantially enhanced the classification outcomes compared to scenarios without any prediction model. Among these models, the use of CNN and fusion models exhibited superior clinical benefits for automatic classification. Figure 7B depicts the DCA curves for different models in the test cohort.

### Performance comparison with junior radiologists

**Fig. 8:**
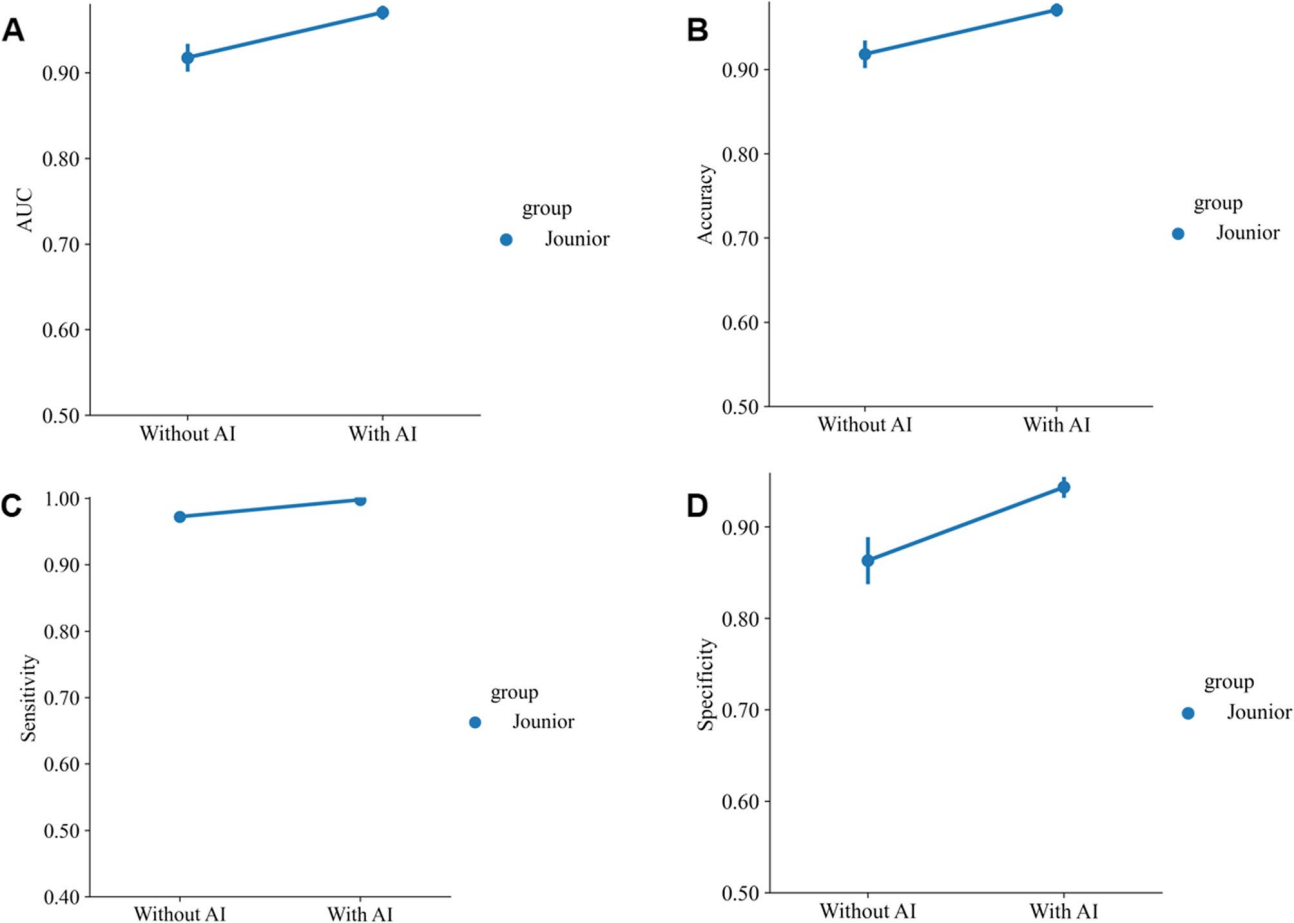
The average AUC, accuracy, sensitivity, and specificity of the junior radiologists without and with the assistance of the AI model

Compared to the feature fusion model (AI model), junior radiologists exhibited lower classification performance in test cohort (average AUC: 0.921, accuracy: 92.2%, sensitivity: 97.2%, specificity: 87.0%, PPV: 88.4%, NPV: 96.8%, precision: 88.4%). Moreover, with the assistance provided by the AI model, junior radiologists significantly enhanced their classification performance (average AUC: 0.971, accuracy: 97.1%, sensitivity: 99.8%, specificity: 94.3%, PPV: 94.7%, NPV: 99.7%, precision: 94.7%). Table 6 presents the evaluation indices of the junior radiologists without and with the assistance of the AI model. Figure 7C and D respectively illustrate the ROC curves for junior radiologists without and with the assistance of the AI model. Figure 8 displays the average AUC, accuracy, sensitivity, and specificity of the junior radiologists without and with the assistance of the AI model.

**Table 6:** Classification performance of junior radiologists without and with the assistance of the AI model

Group	Cohort	AUC (95% CI)	Accuracy (%)	Sensitivity (%)	Specificity (%)	PPV (%)	NPV (%)	Precision (%)
AI	Feature fusion	0.978 (0.965–0.990)	93.2	93.1	93.4	93.5	93.0	93.5
Junior radiologist 1	Without AI	0.932 (0.908–0.955)	93.2	97.7	88.7	89.8	97.4	89.8
Junior radiologist 2	Without AI	0.904 (0.876–0.931)	90.4	96.8	84.0	86.0	96.2	86.0
Junior radiologists Average	Without AI	0.918	91.8	97.2	86.3	87.9	96.8	87.9
Junior radiologist 1	With AI	0.976 (0.962–0.991)	97.7	100.0	95.3	95.6	100.0	95.6
Junior radiologist 2	With AI	0.965 (0.947–0.982)	96.5	99.5	93.4	93.9	99.5	93.9
Junior radiologists Average	With AI	0.971	97.1	99.8	94.3	94.7	99.7	94.7

### Interpretability and visualization of feature fusion model

**Fig. 9:**
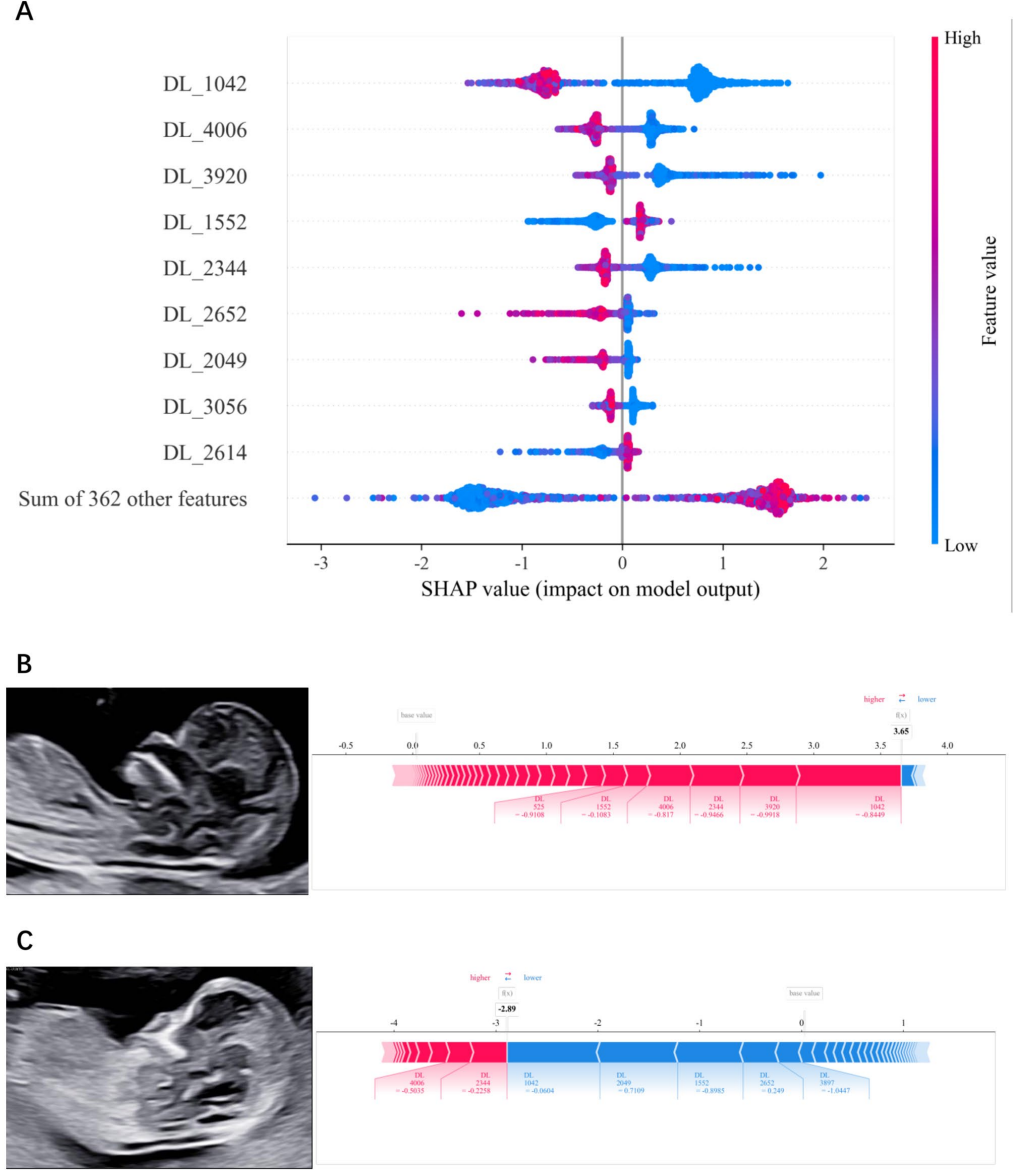
**A **The SHAP summary plot of the feature fusion model; **B** The SHAP force plot of a standard section: the final SHAP value for this image was 3.65, which was larger than the base value of 0.02, thereby indicating that this image was classified as a standard section by the feature fusion model; **C** The SHAP force plot of a non-standard section: the final SHAP value for this image was − 2.89, lower than the base value of 0.02, thereby indicating that this image was classified as a non-standard section by the feature fusion model. DL: deep learning feature

The SHAP method offers interpretability and visualization for the feature fusion model (using MLP algorithm) via the SHAP summary plot and SHAP force plot. The SHAP summary plot displays the significance of features to the model predictions via SHAP values (Fig. 9A). Features are ranked according to their importance, with more important features appearing higher in the summary plot. Each dot, representing a sample, is plotted horizontally. The x-axis shows the SHAP value of the corresponding feature for each dot. The greater the absolute value of the SHAP value, the wider the dot’s distribution from the centerline, indicating a more significant impact of the feature on the prediction results. The color of each dot corresponds to the feature’s contribution to the model prediction (positive or negative).

The SHAP force plot interprets and visualizes the SHAP values of the features for an individual image and their contributions to the model’s prediction results (Fig. 9B and C). The contribution of an individual feature is represented by a colored arrow. The length of the arrow indicates the extent of the feature’s impact on the final prediction (the longer the arrow, the larger the SHAP value, the greater the contribution), and the color of the arrow signifies whether the feature’s contribution to the prediction value is positive (red) or negative (blue). The final SHAP value represents the model’s ultimate prediction for the image, which is the sum of the base value and the cumulative SHAP values from all features. If the final SHAP value is larger than base value, the image is classified as a standard section.

## Discussion

### Main findings

In this study, we constructed and validated radiomics, DTL, and fusion models for the intelligent quality assessment of ultrasound images for fetal NT measurement during the first trimester of pregnancy. All of these models achieved commendable classification performance. Among them, the feature fusion model, which integrated radiomics, CNN, and ViT features to select the most relevant features, demonstrated the best performance in test cohort. It can accurately and automatically classify ultrasound images into standard sections and non-standard sections. More importantly, this model exhibited superior classification performance compared to the junior radiologists and can assist them in enhancing their professional skills. Additionally, we developed an automatic image segmentation model to replace manual annotation, achieving a very high level of segmentation accuracy and significantly saving labor and time.

### Interpretation of results

To find the best classification model, we established and compared radiomics, CNN, ViT, feature fusion, and signature fusion models. The results indicated that the CNN models outperformed radiomics and ViT models with statistical significance (*P* < 0.05). CNN is a computational algorithm inspired by biological neural networks and consists of multilayered artificial neurons between input and output [30]. It is adept at processing vast amounts of data in a layered, non-linear fashion, employing pattern recognition to extract highly representative image features that ascertain the relationship between inputs and labels [17]. It has rapidly become a preferred medical image analysis method [31]. However, CNNs are incapable of capturing the contextual relationships between image features within a global context. In contrast, the recently emerged ViT was introduced to address the image processing limitations of conventional machine learning architectures [18]. This model applies a self-attention mechanism to extract the global relationships between features [19]. Nevertheless, it exhibits limitations in feature localization compared with CNNs [18]. In this study, we proposed two fusion models (early fusion and late fusion) that integrated the advantages of both CNN and ViT models to enhance performance. The results also confirmed the effectiveness of the fusion strategy. In addition, the CNN-based segmentation model achieved satisfactory segmentation accuracy, which can replace manual delineation and greatly improve the efficiency of the model. This may be attributed to the typical presence of amniotic fluid around the fetus, and the ultrasound echoes of the fetus and amniotic fluid are completely different, forming a strong contrast.

Numerous complex machine learning algorithms, especially deep learning architectures, pose significant challenges in interpretation and exhibit a distinctive black-box nature. The lack of interpretability and visualization significantly impedes the widespread adoption of AI models [32]. In this work, we used the SHAP method to interpret and visualize the feature fusion model, providing a clinician-friendly illustration of the importance of features and their impact on the overall prediction model [33]. The SHAP summary plot shows how the features influenced the model’s outputs, as indicated by the range and color of the dots representing the SHAP values and feature values. It can be discerned that the DTL features were identified as the most important features for the feature fusion model. Furthermore, the SHAP force plot enables us to comprehend the contributions of the features for each individual sample to the prediction results.

### Implications for clinical practice

Obtaining the standard midsagittal section of the fetal face is crucial for ensuring accurate and reproducible NT measurement for screening the risk of aneuploidy. However, due to suboptimal fetal positioning or a shortage of trained sonographers, NT measurements are sometimes performed on non-standard sections in clinical practice, resulting in imprecise NT measurement and consequently affecting the evaluation of fetal abnormality risk. Therefore, it is important to perform quality assessment of ultrasound images. As manual quality assessment is time-consuming and laborious, the AI model developed in this study holds substantial clinical application potential. It can replace manual quality assessment, reduce the workload of radiologists, and optimize clinical workflow, especially in resource-constrained settings. Moreover, the model has the potential to assist in training and improving the professional skills of less experienced radiologists. In this study, we utilized the SHAP method to interpret and visualize the model’s decision-making process. The SHAP force plot enables us to understand the contributions of the features to the classification result for each image through SHAP values. In cases of difficulty or uncertainty in classification, junior radiologists can refer to the model’s classification and the analysis of SHAP force plots to identify which features (radiomics or deep learning features) were most important in the classification process, thereby adjusting their classifications accordingly.

### Comparison with previous studies

Several previous studies have developed various AI models for evaluating the midsagittal ultrasound sections of the fetal face, primarily concentrating on automated detection of anatomical regions or standard sections and automatic measurement [21–25]. In comparison, a limited number of studies have focused on the quality assessment and automatic segmentation of standard sections. Lin et al. [34] proposed a deep learning model, Fetus Framework, designed to identify nine key intracranial structures during early pregnancy and further employed for the classification of standard/non-standard sections. The results revealed that intracranial structures identified by the Fetus Framework aligned perfectly with those determined by senior radiologists. The model achieved an AUC of 0.974 (95%CI: 0.952, 0.995) in external test to classify standard/non-standard sections and outperformed junior radiologists. Xue et al. [35] proposed a detection network for the identification and quality assessment of standard sections of fetal facial in early pregnancy. This model automatically scored the key anatomical structures adhering to a control protocol, determining whether they were standard sections. The results demonstrated this model performed well, with a precision rate of 97.20% for the classification. The performance of the fusion models in our study is comparable to the outcomes of previous studies.

### Strengths and limitations

To the best of our knowledge, few studies have applied the ViT model to analyze fetal ultrasound images. We innovatively developed an early fusion model that integrated the radiomics, CNN, and ViT features for intelligent quality assessment in NT measurement, achieving satisfactory classification performance. The proposed models address two gaps in prior studies: (1) Establishing a deep learning-based image segmentation model to automatically delineate the ROI of ultrasound images for NT measurement. (2) Developing a fusion model that combined the advantages of radiomics, CNN, and ViT models for classifying images in NT measurement into standard and non-standard sections.

However, the limitations are as follows: (1) This study was conducted retrospectively with a limited sample size and excluded multiple pregnancies and severely abnormal fetuses, which may have introduced sample selection bias. (2) This was a two-center study, which limited the generalizability of the model. In the future, multi-center prospective researches with larger sample sizes are needed to confirm the performance of the model and enhance its generalizability. Additionally, future research directions include developing an AI model for scoring sections based on the recognition of different anatomical structures, conducting further research on more comprehensible methods for model visualization and interpretation, and incorporating advanced techniques such as contrastive learning or generative methods to enhance model performance [36, 37].

## Conclusions

The proposed models innovatively bridge the gaps in previous studies, achieving intelligent quality assessment of ultrasound images for NT measurement and highly accurate automatic segmentation of ROIs. The successful fusion strategy of the feature fusion model, which integrates the advantages of radiomics, CNN, and ViT models, achieves commendable classification performance. These AI models are potential tools to enhance quality control in ultrasound examinations, streamline clinical workflows, partially supplant manual quality assessments, and elevate the professional skills of less experienced radiologists.

## Supplementary Information

Supplementary Material 1.

## Data Availability

The data that support the findings of this study are available on request from the corresponding author (P.Y.). The data are not publicly available due to privacy or ethical restrictions.

## Abbreviations

NT: Nuchal translucency
ROI: Region of interest
DTL: Deep transfer learning
SHAP: SHapley Additive exPlanations
ISUOG: International society of ultrasound in obstetrics and gynecology
AI: Artificial intelligence
CNN: Convolutional neural network
ViT: Vision transformer
ICC: Interclass correlation coefficient
DSC: Dice similarity coefficient
HD 95: 95th percentile Hausdorff distance
LASSO: Least absolute shrinkage and selection operator
LR: Logistic regression
SVM: Support vector machine
KNN: k-nearest neighbor
MLP: Multi-layer perception
ROC: Receiver operating characteristic
AUC: Area under the curve
PPV: Positive predictive value
NPV: Negative predictive value
DCA: Decision curve analysis
SD: Standard deviation
CI: Confidence interval
MSE: Mean square error
