# Supplementary material for: Intelligent quality assessment of ultrasound images for fetal nuchal translucency measurement during the first trimester of pregnancy based on deep learning models
Source: BMC Pregnancy Childbirth. 2025 Jul 10;25:741. doi: 10.1186/s12884-025-07863-y (PMC12243185; doi:10.1186/s12884-025-07863-y)
Supplement: Supplementary file 1 — Supplementary Material 1. [file 12884_2025_7863_MOESM1_ESM.doc]

- **Hyperparameter details for the machine learning models:**

1. **Logistic Regression (LR)**

Regularization penalty is disabled (penalty='none')

Maximum training iterations set to 100 (max_iter=100)

1. **Support Vector Machine (SVM)**

Linear kernel function is used (kernel='linear')

Probability estimation is enabled (probability=True)

Maximum solver iterations limited to 100 (max_iter=100)

1. **Random Forest**

Number of decision trees set to 5 (n_estimators=5)

Maximum tree depth restricted to 3 levels (max_depth=3)

Minimum 4 samples required to split a node (min_samples_split=4)

Random seed fixed at 0 (random_state=0)

1. **XGBoost**

Boosting rounds limited to 6 (n_estimators=6)

Binary logistic regression objective function (objective='binary:logistic')

Tree depth capped at 3 levels (max_depth=3)

Minimum sum of instance weight in leaf nodes set to 0.2 (min_child_weight=0.2)

Label encoder usage is disabled (use_label_encoder=False)

Evaluation metric uses classification error rate (eval_metric='error')

1. **LightGBM**

Boosting iterations limited to 2 (n_estimators=2)

Tree depth constrained to 1 level (max_depth=1)

Minimum sum of instance weight in leaves set to 0.5 (min_child_weight=0.5)

1. **Extra Trees**

Ensemble contains 60 decision trees (n_estimators=60)

Maximum tree depth limited to 5 levels (max_depth=5)

Minimum 2 samples required for node splitting (min_samples_split=2)

Random seed fixed at 0 (random_state=0)

1. **Gradient Boosting**

Boosting stages limited to 10 (n_estimators=10)

Random seed fixed at 0 (random_state=0)

1. **AdaBoost**

Number of weak learners set to 10 (n_estimators=10)

Random seed fixed at 0 (random_state=0)

1. **Multilayer Perceptron (MLP)**

Network architecture contains four hidden layers with 61, 128, 64, and 32 neurons respectively (hidden_layer_sizes=(61,128,64,32))

Training capped at 300 epochs (max_iter=300)

Stochastic gradient descent optimizer is used (solver='sgd')

Random seed fixed at 0 (random_state=0)

- **Hyperparameter details for the deep learning model (VGG19_bn):**

1. **Data Configuration**

Training dataset loaded from train_f (train=train_f)

Validation dataset loaded from val_f (valid=val_f)

Class labels sourced from labels_f (labels_file=labels_f)

Input data pattern defined by data_pattern (data_pattern=data_pattern)

1. **Resource Management**

Parallel processing uses 2 threads (j=2)

No limit on training samples (max2use=None)

No limit on validation samples (val_max2use=None)

GPU device 0 selected for training (gpus=[0])

1. **Training Setup**

Batch balancing disabled (batch_balance=False)

ImageNet-standard normalization applied (normalize_method='imagenet')

Mini-batch size set to 32 (batch_size=32)

Total training epochs: 50 (epochs=50)

Initial learning rate: 0.01 (init_lr=0.01)

Optimization algorithm: SGD (optimizer='sgd')

1. **Model Architecture**

Backbone model: VGG19 with Batch Normalization (model_name='vgg19_bn')

Vision Transformer (ViT) settings:

- Patch size: 64 × 64 pixels (patch_size=64)
- Embedding dimension: 1024 (dim=1024)
- Transformer depth: 6 layers (depth=6)
- Attention heads: 16 (heads=16)
- MLP hidden dimension: 2048 (mlp_dim=2048)

Pretrained weights initialization enabled (pretrained=True)

1. **Logistics & Output**

Model outputs saved to current directory (model_root='.')

Timestamps excluded from output filenames (add_date=False)

Training starts from iteration 0 (iters_start=0)

Progress logging frequency: every iteration (iters_verbose=1)

Epoch-level checkpoint saving disabled (save_per_epoch=False)

No retraining path specified (retrain=None)

- **Hyperparameter details for the deep learning model (ViT):**

**1. Data Pipeline**

Training data path: train_f

Validation data path: val_f

Label file path: labels_f

Input data pattern: data_pattern

No maximum sample limit for training/validation (max2use=None, val_max2use=None)

Batch balancing disabled (batch_balance=False)

ImageNet standardization applied (normalize_method='imagenet')

**2. Computational Resources**

GPU device ID 0 utilized (gpus=[0])

Parallel processing threads: 2 (j=2)

**3. Training Protocol**

Batch size: 32 samples (batch_size=32)

Total training epochs: 50 (epochs=50)

Initial learning rate: 0.01 (init_lr=0.01)

Stochastic Gradient Descent optimizer (optimizer='sgd')

Training resumes from iteration 0 (iters_start=0)

Progress logging frequency: Every iteration (iters_verbose=1)

**4. Vision Transformer Architecture**

Base model: Vision Transformer (model_name='ViT')

Transformer configuration:

Input patch size: 64×64 pixels (patch_size=64)

Embedding dimension: 1024 (dim=1024)

Transformer blocks: 6 layers (depth=6)

Multi-head attention heads: 16 (heads=16)

MLP hidden dimension: 2048 (mlp_dim=2048)

Pretrained weights initialization enabled (pretrained=True)

**5. Model Management**

Model output directory: Current folder (model_root='.')

No timestamp in output filenames (add_date=False)

Epoch-level model saving disabled (save_per_epoch=False)

Retraining path unspecified (retrain=None)
